# The impact of criminalization of HIV non-disclosure on the healthcare engagement of women living with HIV in Canada: a comprehensive review of the evidence

**DOI:** 10.7448/IAS.18.1.20572

**Published:** 2015-12-22

**Authors:** Sophie E Patterson, M-J Milloy, Gina Ogilvie, Saara Greene, Valerie Nicholson, Micheal Vonn, Robert Hogg, Angela Kaida

**Affiliations:** 1 Faculty of Health Sciences, Simon Fraser University, Burnaby, BC, Canada; 2 BC Centre for Excellence in HIV/AIDS, St Paul's Hospital, Vancouver, BC, Canada; 3 Faculty of Medicine, University of British Columbia, Vancouver, BC, Canada; 4 School of Social Work, McMaster University, Hamilton, ON, Canada; 5 British Columbia Civil Liberties Association, Vancouver, BC, Canada

**Keywords:** HIV, criminalization, HIV non-disclosure, women, Canada

## Abstract

**Introduction:**

In 2012, the Supreme Court of Canada ruled that people living with HIV (PLWH) must disclose their HIV status to sexual partners prior to sexual activity that poses a “realistic possibility” of HIV transmission for consent to sex to be valid. The Supreme Court deemed that the duty to disclose could be averted if a person living with HIV both uses a condom *and* has a low plasma HIV-1 RNA viral load during vaginal sex. This is one of the strictest legal standards criminalizing HIV non-disclosure worldwide and has resulted in a high rate of prosecutions of PLWH in Canada. Public health advocates argue that the overly broad use of the criminal law against PLWH undermines efforts to engage individuals in healthcare and complicates gendered barriers to linkage and retention in care experienced by women living with HIV (WLWH).

**Methods:**

We conducted a comprehensive review of peer-reviewed and non-peer-reviewed evidence published between 1998 and 2015 evaluating the impact of the criminalization of HIV non-disclosure on healthcare engagement of WLWH in Canada across key stages of the cascade of HIV care, specifically: HIV testing and diagnosis, linkage and retention in care, and adherence to antiretroviral therapy. Where available, evidence pertaining specifically to women was examined. Where these data were lacking, evidence relating to all PLWH in Canada or other international jurisdictions were included.

**Results and discussion:**

Evidence suggests that criminalization of HIV non-disclosure may create barriers to engagement and retention within the cascade of HIV care for PLWH in Canada, discouraging access to HIV testing for some people due to fears of legal implications following a positive diagnosis, and compromising linkage and retention in healthcare through concerns of exposure of confidential medical information. There is a lack of published empirical evidence focused specifically on women, which is a concern given the growing population of WLWH in Canada, among whom marginalized and vulnerable women are overrepresented.

**Conclusions:**

The threat of HIV non-disclosure prosecution combined with a heightened perception of surveillance may alter the environment within which women engage with healthcare services. Fully exploring the extent to which HIV criminalization represents a barrier to the healthcare engagement of WLWH is a public health priority.

## Introduction

In many settings worldwide, there is a reliance on criminal prosecutions of HIV transmission, exposure and non-disclosure in efforts to reduce HIV incidence [1, 2]. Human rights advocates and public health scientists have condemned these prosecutions, maintaining that the use of the criminal law against people living with HIV (PLWH) jeopardizes public health efforts to meet their health needs [3–11] and further complicates gendered barriers to linkage and retention in HIV care [3, 12–18]. We reviewed the evidence to determine how the threat of HIV non-disclosure prosecution affects healthcare engagement of women living with HIV (WLWH) in Canada, one of the countries that most aggressively uses the criminal law against PLWH [1].

### Canadian legal precedent for HIV non-disclosure prosecutions

**Figure 1:**
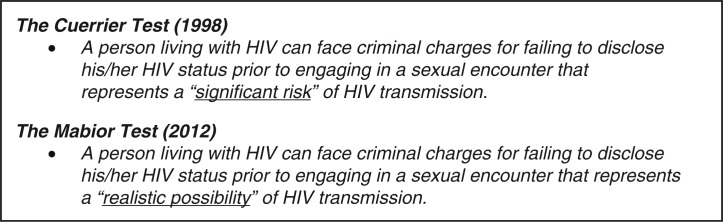
Summary of the historical and current case law for HIV non-disclosure, reflecting two key rulings by the Supreme Court of Canada [19, 22, 23].

Since the late 1980s, PLWH in Canada have faced the risk of criminal charges if they did not disclose their HIV status before a sexual encounter. In 1998, the matter came before the Supreme Court of Canada (SCC) in R. v. Cuerrier (“Cuerrier”), which found that there was a duty to disclose when sexual activity presented a “significant risk” of transmitting HIV (Figure 1) [19]. In this case, an HIV-positive man in British Columbia was charged with aggravated assault after allegedly failing to disclose his HIV status before condomless sexual intercourse with two women. Since Cuerrier, criminal charges have been brought on the basis of HIV non-disclosure, regardless of whether or not HIV transmission occurred, or whether intent to transmit was established. The SCC's ruling in Cuerrier left many scenarios to be determined with respect to the “significant risk” threshold. For example, the relevance of condom use to reduce the risk of HIV transmission was not settled in Cuerrier, and application and interpretation of the law varied across jurisdictions and cases in the following years. However, in at least four criminal cases after Cuerrier, it was judged that “significant risk” of HIV transmission (and legal duty to disclose) was averted if a condom was used [20, 21].

In October 2012, the SCC set a new and more rigorous test for Canadian HIV non-disclosure prosecutions in its rulings in R v. Mabior and R v. DC [22, 23]. The court ruled that PLWH who do not disclose their HIV status before a sexual activity that poses a “realistic possibility” of HIV transmission can face criminal charges for aggravated sexual assault (Figure 1). The court ruled that in circumstances where condom-protected penile-vaginal intercourse occurred with a low viral load (<1500 copies/mL), the realistic possibility of HIV transmission would be negated, and criminal liability for non-disclosure would be avoided [22, 23]. However, the court left it unclear whether this reasoning would apply to other sexual acts besides vaginal sex. The SCC found that HIV non-disclosure prior to sex that posed a realistic possibility of HIV transmission constituted fraud that vitiated the consent to sexual activity. A conviction can result in a maximum sentence of life imprisonment and mandatory listing on the National Sex Offender Registry.

Canada has one of the strictest legal thresholds for criminal prosecutions of PLWH globally and has produced the second highest absolute number of convictions among individuals charged with HIV non-disclosure, after the United States [1]. The Canadian legal approach is contrary to national and international recommendations [24–27]. Moreover, the legal interpretation of HIV transmission risk fails to reflect current scientific evidence [27, 28]. The probability of HIV transmission from a WLWH who is not on antiretroviral therapy (ART) to a seronegative male partner is estimated at 0.04% per act of condomless penile–vaginal intercourse, with the transmission risk significantly elevated in early and late stages of HIV infection [29]. Condoms reduce the probability of HIV transmission during penile–vaginal intercourse by an estimated 80% [30]. The use of ART by PLWH further reduces the risk of HIV transmission to sexual partners. The Swiss Federal AIDS Commission released a landmark statement in 2008, which stated that PLWH who are adherent to ART for six months, with an undetectable viral load (<40 copies/ml) and no concurrent sexually transmitted infections, could not transmit HIV through sexual contact [31]. The negligible possibility of HIV transmission per act of condomless penile–vaginal intercourse associated with ART adherence has been further supported by contemporary studies and expert commentaries from respected researchers and clinicians [27, 32–36].

Between 1989 and October 2015, an estimated 184 individuals were charged for HIV non-disclosure in Canada [E Mykhalovskiy, personal communication]. The use of the criminal law against PLWH has increased since the late 1980s [20, 37, 38], with notable increases in the annual number of charges following the release of key rulings from the SCC [37]. Most charges have been brought against heterosexual men, with African/Black men disproportionately represented [37, 39, 40]. Women account for a quarter of incident HIV cases in Canada annually [41], while female defendants have featured in approximately 10% of non-disclosure prosecutions [21]. Notably, however, marginalized women are overrepresented among the 17 women who have been charged, including sex workers, women living with addiction, survivors of abuse, and Indigenous women [12, 21, 42]. The fact that women have more frequently represented the complainants in HIV non-disclosure criminal cases to date may reflect the fact that the Canadian criminal justice system treats HIV non-disclosure as a sexual offence, triggering preconceptions about expected gender identities of complainant and defendant [21, 37], further fuelled by inflammatory media reports of criminal cases with male defendants [43–45].

### Historical considerations

There is a critical need to consider HIV criminalization through a gendered lens. Across a diversity of global settings, women experience gender-based inequities, including relationship power imbalance, intimate partner violence and a subordinate legal status, which increase HIV acquisition risks [46]. In the late 1980s, laws criminalizing HIV non-disclosure, transmission and exposure were viewed and pursued as a means of protecting women from HIV acquisition [47]. However, in the years since, women's advocates have argued that HIV criminalization is a blunt tool, and an ineffective method of HIV prevention among women [12].

In recent Canadian history, progressive sexual assault laws were achieved, along with an affirmative, robust definition of consent, following a hard-fought campaign for women's equality, dignity and sexual autonomy [48, 49]. These laws were intended to empower women's autonomous sexual decision-making, including demanding consensual and safer sexual practices [49]. The fact that sexual assault laws are being used in Canada to prosecute cases of HIV non-disclosure among women they originally sought to protect is contrary to the spirit of this women's rights movement [12, 21, 48]. Far from promoting an individual's responsibility and right to protect themself, scholars and human rights advocates emphasize that HIV criminalization endorses messaging that safe sex and HIV prevention is the exclusive (and legal) responsibility of PLWH [12, 47] and contributes to the portrayal of PLWH as “reckless vectors” and their sexual partners as innocent victims, driving social anxieties and misconceptions around HIV, and failing to advance gender equality [50].

### Women and the criminalization of HIV non-disclosure

There are an estimated 16,600 WLWH in Canada [51], with overrepresentation from members of marginalized subpopulations, including Indigenous women, women who use injection drugs, sex workers, immigrant and refugee women, and LGBTQ women [52, 53]. WLWH may be differently affected by environments shaped through the criminalization of HIV non-disclosure as compared to men. Many women receive routine HIV testing through antenatal health services and are thus more likely to be aware of their positive HIV status [12, 54]. Although gender differences in disclosure rates are inconsistently observed across international studies, the literature is consistent regarding women's unique barriers to and consequences of HIV disclosure [55]. Women may delay disclosure to sexual partners due to fears of stigma, discrimination, social isolation and rejection [55–58]. In particular, women who face power inequality within dependent partnerships may risk violence or abandonment associated with disclosing their status, insisting on condom use, or refusing sexual advances [56, 59–62] and may be less likely to satisfy the Canadian legal test for HIV non-disclosure [63, 64].

A concern is that the threat of HIV criminalization may jeopardize public health initiatives focused on addressing the health needs of WLWH [3–11]. Evidence suggests that WLWH in Canada experience delayed access to HIV care [65, 66]; poorer initial quality of HIV care [67]; increased risk of treatment interruptions [68]; and poorer treatment outcomes, in terms of ART adherence [69, 70], viral suppression [71–74] and viral rebound [72, 75], compared to men. The overly broad use of the criminal law against PLWH in Canada combined with inflammatory media reporting of criminal cases [76, 77] contributes to a surveillance environment that fosters uncertainty, fear and vulnerability among PLWH [78–81]. Consequently, HIV criminalization in Canada may represent an additional barrier to the healthcare engagement of WLWH [12, 54].

## Aim of review

**Figure 2:**
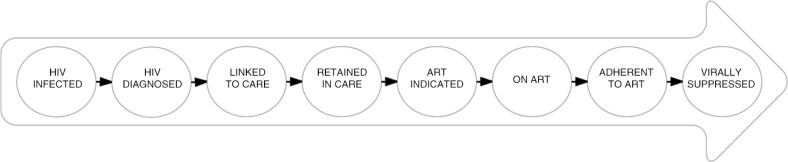
Gardner's cascade of HIV care. Figure illustrating key steps in the cascade of HIV care, from primary HIV infection to viral suppression [82].

Current best practice in HIV care is to meaningfully engage PLWH in HIV care services to optimize individual and public health benefits of ART. Increasingly, this is conceptualized within the cascade of HIV care [82] (Figure 2). The cascade outlines incremental stages of engagement with HIV treatment and care required to achieve viral suppression, and is used to monitor the success of HIV care initiatives [73, 83]. The strategy known as Treatment-as-Prevention (TasP) aims to promote high levels of viral suppression through ART use and retention in the cascade of HIV care, to both curb HIV-related morbidity and mortality and reduce onward HIV transmission [84–86]. However, a crucial challenge for the success of TasP campaigns is addressing social and structural level barriers to optimal engagement in the cascade [87]. We sought to determine the effect of the criminalization of HIV non-disclosure on healthcare engagement of Canadian WLWH across the cascade of HIV care.

## Methods

We performed a comprehensive review of peer-reviewed and non-peer-reviewed literature to evaluate the impact of HIV non-disclosure criminalization on the engagement of Canadian WLWH across key stages of the cascade of HIV care, specifically: HIV testing and diagnosis, linkage and retention in care, and ART access and adherence. Our search was limited to literature written in the English language and published between December 1998 and September 2015. Where available, literature pertaining specifically to Canadian WLWH was examined. Where lacking, literature relating to other Canadian populations living with HIV was reviewed. Inclusion of literature was limited to Canadian studies due to the specificity of Canadian HIV non-disclosure case law and healthcare delivery systems. Literature from international jurisdictions was included only when Canadian literature was lacking.

We commenced the literature search in PubMed, using the search terms: HIV law; HIV criminalization; HIV non-disclosure; HIV law women; HIV law Canada; HIV law public health; HIV law testing; HIV law antiretroviral therapy; HIV law healthcare engagement; HIV law adherence. Duplicate searches were completed using Google Scholar and Simon Fraser University's online library to identify missed publications. We reviewed the reference lists of retrieved articles to identify articles missed by our search strategy. We reviewed titles of abstracts presented at the International AIDS Conference (AIDS); the International AIDS Society Conference on HIV Pathogenesis, Treatment and Prevention; and the Canadian Association for HIV/AIDS Research conference to identify presentations relevant to the aims of this review. Abstracts of potentially relevant articles were read to confirm suitability for inclusion in the review, before a detailed review was completed. Identified literature was categorized into three key topics: HIV diagnosis and testing; linkage and retention in HIV care; and access and adherence to ART. To contextualize our research findings, we reviewed relevant clinical guidelines and literature published by HIV organizations, including the Canadian HIV/AIDS Legal Network.

## Results and discussion

We identified 20 articles based on 16 Canadian studies that presented data on the impact of HIV criminalization on healthcare engagement of PLWH, two of which specifically focused on WLWH. Canadian studies evaluating the impact of HIV criminalization on access and adherence to ART were lacking. This required expanding our search to other settings, which identified an additional three articles based on two studies (Appendix: Table 1).

### HIV diagnosis and testing

HIV testing is the first stage of engagement within the cascade of HIV care, when people with HIV are diagnosed and subsequently linked with health services [82]. In Canada, an estimated 25% of PLWH are unaware of their HIV serostatus [41]. Increasing HIV testing among individuals who suspect that they may be HIV-positive but do not wish to know their status [88, 89] and those truly unaware of their status is a rate limiting step in the cascade of HIV care, compromising the ability to identify and link to care those most at risk of onward HIV transmission [90].

Several Canadian studies have evaluated the perceived impact of HIV criminalization on HIV testing practices. In qualitative interviews and focus groups with WLWH [91], healthcare providers [92] and stakeholders working with PLWH [93], participants expressed the opinion that HIV criminalization negatively affects willingness to test for HIV. However, these qualitative data did not capture perspectives of people who may be personally deterred from accessing HIV testing in the current legal climate [91–93]. In a qualitative study among 27 gay, bisexual and other men who have sex with men (MSM), HIV-negative participants believed that fear of non-disclosure prosecutions reduced the willingness to access HIV testing in the community [94]. Similarly, in two national cross-sectional surveys conducted in 2011 among 2139 Canadians (52% female) and 1235 MSM (67% HIV-negative), 31% and 48% of participants believed that criminal prosecutions reduced willingness to access HIV testing, respectively [88, 95]. However, in these analyses, participants did not report on whether their own testing practices were affected by non-disclosure prosecutions.

Studies presenting data on personal HIV testing practices suggest that fear of non-disclosure prosecutions affects HIV testing for some Canadians [96, 97]. In a clinic-based survey among 150 HIV-negative MSM in Toronto, few (7%) participants reported that fears of prosecution made them less likely to access HIV testing [98]. However, in a survey of 721 gay, bisexual and MSM (85% HIV-negative) in Ottawa, 21% of participants reported that the risk of HIV non-disclosure prosecutions negatively affected their decision to access HIV testing [96]. Notably, among HIV-negative and unknown status participants, those reporting that non-disclosure prosecutions affected their testing practices were less likely to have previously received an STI/HIV test and more likely to report a preference for anonymous HIV testing [97, 98], representing a key target group for HIV testing initiatives. Anonymous testing has the highest HIV-positivity rate of all testing services in Ontario [99], which may suggest that these participants suspect that they are HIV-positive but willingness to test through standard testing is negatively affected by fear of prosecution. In contrast, in qualitative interviews among PLWH in Ontario, participants reported that non-disclosure prosecutions did not influence their HIV testing practices prior to their HIV diagnosis [79]; however, most participants were tested before the incidence of Canadian criminal prosecutions increased (from 2004 onwards) [37].

To our knowledge, only one analysis has used Canadian population-based HIV testing rates to assess an association between HIV testing and HIV criminalization. This assessment of regional HIV testing rates among MSM in Ottawa revealed no significant decrease in testing rates after media coverage of a local, high-profile non-disclosure prosecution in 2010 [94]. However, HIV testing decisions may have been influenced by a variety of competing factors, including health status, which may diminish the ability to detect an impact of non-disclosure prosecutions on testing practices [94].

The expansion of HIV testing to identify the undiagnosed population living with HIV and reach ambitious 90-90-90 UNAIDS treatment targets [100] is a national public health priority [66]. The literature reviewed here offers some evidence that HIV criminalization may introduce an additional structural level barrier to HIV testing for some individuals, possibly those who anticipate a positive result. Even if a minority of individuals are deterred from HIV testing, this may compromise the ability to meet the UNAIDS target that 90% of PLWH should know their HIV status by 2020 [100]. Establishing a clear evidence-based association between HIV testing and the criminal law presents significant challenges due to the myriad of divergent individual, social and structural factors, which interplay to affect HIV testing decisions.

Our review revealed a dearth of studies specifically evaluating the impact of HIV criminalization on HIV testing among Canadian women. As a result of routine antenatal HIV testing, the majority of WLWH in Canada are tested for HIV during pregnancy. In Ontario, the province with the largest number of WLWH in Canada, 96% of pregnant women were tested for HIV prenatally in 2010, with 18 HIV-positive diagnoses (0.13 per 1000) [101]. Opt-out antenatal HIV testing protocols are operational in the majority of Canadian provinces and territories [102], with the important aim of increasing uptake of HIV testing, and ensuring early ART initiation to prevent mother-to-child transmission [103]. However, HIV testing circumstances may influence decisions to disclose and engage with health services. Women accessing HIV testing in traditional risk-based, client-initiated voluntary counselling and testing settings make a considered, risk-based decision to present for HIV testing, often after discussion with their partner [56]. However, women who are offered routine HIV testing in a prenatal clinic appointment may not have previously considered accessing testing, and may face additional barriers to accepting their positive diagnosis [104], engaging with treatment services or disclosing to partners [56].

Canadian antenatal HIV testing guidelines from 2006 recommend that testing during pregnancy should be voluntary, with women reserving the right to refuse testing after receiving comprehensive counselling [105]. The 2012 Canadian HIV Pregnancy Planning guidelines state that women testing positive should be further counselled about the legal implications of HIV non-disclosure [106]. However, routine opt-out testing protocols may compromise the counselling and consent process [107]; limiting pre-test counselling to convey the potential legal implications of a positive result, and removing the opportunity to refuse testing or request anonymous testing [108–111]. In qualitative interviews exploring experiences of opt-out antenatal HIV testing among 12 pregnant women in Newfoundland and Labrador, no participants were advised they had the right to refuse HIV testing, and some participants were tested for HIV without providing formal consent or being aware that they were being tested [112]. This qualitative study raised concerns that an opt-out approach to testing may threaten provider trust, and affect future health seeking behaviour [112]. Similarly, in a survey administered to 299 postpartum women in Toronto, 74% of participants reported receiving pre-test counselling before antenatal HIV testing, and 70% of these participants were given the option to refuse the test [113]. These findings are concerning in the climate of criminalization, when failure to provide comprehensive pre-test counselling and acquire informed consent may not only pose a threat to civil liberties, autonomy and privacy of information [111], but may also limit awareness of the legal obligation to disclose.

### Linkage and retention in HIV care

After receiving an HIV diagnosis, PLWH should be linked with and retained in appropriate care services to ensure optimal health outcomes. Medical confidentiality is vital to encourage patient candidness during clinical consultations and preserve public confidence in the medical system [114]. In Canada, medical confidentiality is legally protected; however, healthcare providers may be obliged to expose confidential health information if called as a witness in a judicial trial or issued with a warrant to produce healthcare records [115]. Similarly, there is precedent for healthcare providers to voluntarily breach confidentiality for reasons of public safety; for example, if they become aware of an immediate risk of serious harm to an identifiable third party [115]. With the 2012 SCC ruling, the relevance of clinical case notes to confirm viral load testimony in court has been confirmed [22, 23], and police and prosecutors have attempted to force disclosure of confidential health documents for use as evidence within judicial trials [116].

Canadian studies suggest that non-disclosure prosecutions can prompt individuals to question the limits of confidentiality in a healthcare setting, resulting in reluctance to engage in open dialogues during clinical consultations, and representing a barrier to linkage and retention in HIV care services. In self-administered anonymous surveys among 721 MSM (85% HIV-negative) in Ottawa in 2012, 15% of the participants reported that non-disclosure prosecutions made them afraid to discuss health concerns with healthcare providers [97]. Participants reporting this fear were more likely to self-report condomless intercourse and multiple sexual partners; individuals most in need of sexual health services [97, 98].

Qualitative interviews among PLWH have also explored the impact of HIV criminalization on healthcare engagement and experience. In semi-structured interviews with 27 HIV-positive and negative MSM in Ottawa in 2012, participants expressed concerns relating to the transfer of health information between the local police and public health departments, resulting in mistrust of healthcare providers [94]. These findings were echoed in semi-structured interviews with African/Black men living with HIV and WLWH in the Greater Toronto Area, during which participants reported experiencing increased stigma and discrimination from healthcare providers due to HIV criminalization, and questioned the privacy of healthcare information [117]. Conversely, in-depth interviews with 122 PLWH in Ontario revealed that participants with longstanding relationships with healthcare providers did not report difficulty trusting their provider in the current legal climate, suggesting that the impact of HIV criminalization on healthcare engagement may depend on the length and quality of pre-established relationships with healthcare providers [79]. Notably, participants were recruited from clinic settings and as such were already engaged with healthcare services, thus may not represent the most marginalized members of the community.

Canadian healthcare providers have similarly voiced concerns that the criminalization of HIV non-disclosure compromises provider-patient relationships [4, 38]. Semi-structured interviews with 25 people who work with PLWH (including lawyers, physicians and counsellors) in Ontario revealed that fear of non-disclosure prosecutions deterred patients from speaking freely with providers about sexual behaviours and disclosure challenges due to anxieties relating to confidentiality of medical documentation [38]. These concerns were reiterated in focus group discussions with 47 service providers, working in nursing, medicine, law and social work in 2011 [118]. Qualitative interviews with 40 PLWH and 15 prevention workers in Toronto identified fear of HIV non-disclosure prosecutions as a deterrent to participating in risk-reduction programs that involved the discussion of sexual history [119]. In semi-structured interviews with 15 HIV/AIDS service providers in Toronto, providers expressed the belief that the SCC's ruling in R v. Mabior increased stigma directed towards PLWH (particularly women, sex workers and those living with addiction), which may compromise healthcare engagement [120].

The climate of criminalization may also influence the clinical practice of healthcare providers, which may in turn compromise the quality of care provided. In semi-structured interviews in Ontario, healthcare providers reported being increasingly mindful of the law when counselling patients in a clinical setting [4, 38]. Similarly, qualitative interviews and focus groups among healthcare workers in the HIV sector have suggested that providers lack understanding about the current legal obligation to disclose [117, 120], which compromises their ability to provide sound counselling to patients [4, 38]. In focus group discussions with 47 service providers in Ottawa, participants expressed concern that disclosure counselling was being approached from a legal rather than healthcare standpoint [118]. Similarly, interviews with 30 public health nurses in Ontario revealed that the risk of subpoena of medical documentation for use in judicial trials influenced patient-provider discussions around the limits of confidentiality in the healthcare setting, with some providers withholding/limiting details about confidentiality to preserve therapeutic relationships [121]. Qualitative data drawn from the latter two studies also suggest that anticipation of possible subpoena of medical documents for use in trials may influence documentary practices within medical records, either to ensure adequate recall of clinical events, to signify professional standards are being upheld, or to maximize patient confidentiality [118, 121, 122].

Our review of the Canadian literature suggests that HIV criminalization may negatively affect healthcare engagement and experiences of PLWH. However, not all individuals report these harmful effects. Previous work suggests that many PLWH are not personally concerned about being prosecuted for HIV non-disclosure [88], particularly individuals who can and do consistently disclose their HIV status, those who are sexually inactive, or those who are in mutually disclosed long-term partnerships [11, 80]. Opinions and experiences of HIV criminalization are diverse and complex [18, 80, 123]. For PLWH, perceptions of HIV criminalization may evolve from the time of initial diagnosis, with some individuals transitioning from the role of “accuser” to “accused” during their life journey [18]. However, the gendered impacts of HIV criminalization on healthcare engagement and experience in Canada have not yet been thoroughly explored.

As the majority (63%) of WLWH in Canada are diagnosed with HIV during childbearing years [124]; most WLWH require reproductive health services as part of their care. In the 2012 Canadian HIV Pregnancy Planning guidelines, the authors express concern that HIV-related prosecutions may affect engagement with reproductive health services, including contraceptive counselling and antenatal programs designed to reduce mother-to-child transmission and promote maternal health outcomes [106]. Although there is a dearth of Canadian studies evaluating the impact of HIV criminalization on engagement with reproductive services, qualitative interviews conducted in Ontario with 77 pregnant WLWH in their third trimester, and at 3 and 12 months postpartum, revealed that women experience increased surveillance and judgment from health and social care providers during pregnancy and early motherhood in the current legal climate [125]. Prosecution of mother-to-child transmission is rare in Canada; however, in 2006 an Ontario mother living with HIV was found guilty of failing to provide the necessities of life to her second child, who acquired HIV after the mother elected not to disclose her HIV status to the medical staff providing her care during childbirth, meaning postpartum antiretrovirals could not be administered to her baby immediately after delivery [126].

The apparent erosion of patient-provider relationships and the negative impact on disclosure counseling in the climate of criminalization are concerning, given that many PLWH demonstrate a critical need for counselling regarding their current legal obligations. Focus group discussions among marginalized HIV-positive and negative female sex workers in Vancouver in 2008 revealed a lack of awareness of the legal obligation to disclose [127]. Poor awareness of the legal obligation to disclose similarly emerged in focus group discussions among 60 WLWH in Vancouver [91], and in qualitative interviews with African/Black men and WLWH in Toronto [117]. In contrast, 91% of HIV-positive participants enrolled in two Ontario-based cohort studies (*n=*930) reported an awareness of the legal obligation to disclose [79]. A highly-educated sample of MSM (over 80% with tertiary education) interviewed in Ottawa in 2012 also demonstrated good (90%) awareness of HIV non-disclosure laws [97]. However, the latter studies are unlikely to be generalizable to the most marginalized PLWH, who are already suboptimally engaged in care. Limited gender-based comparisons of awareness of the legal obligation to disclose among PLWH are available, however in a national survey among 2139 Canadians in 2011, a lower proportion of women reported being aware that PLWH can be prosecuted for HIV non-disclosure (83 vs. 90%, *p*<0.05) [128].

### Access and adherence to ART

For PLWH, optimal adherence to ART is the key determinant of viral suppression [129]. Thus, elements of HIV criminalization that affect access and adherence to ART may limit achievement of viral suppression, resulting in both individual and public health repercussions. Little empirical evidence exists to evaluate the effect of non-disclosure prosecutions on access and adherence to ART in Canada. However, preliminary findings from the National HIV Criminalization Survey, an online survey administered to 2076 PLWH (13% women) in the United States in 2012, revealed that 42% of participants believed it was reasonable to avoid seeking HIV treatment due to concerns relating to the risk of HIV-related prosecutions [130]. No significant differences by gender were identified.

Cross-sectional survey data have been used to demonstrate an association between HIV criminalization and ART adherence [131, 132]. Among 2149 HIV-positive participants (29% women) recruited from 16 sites across Canada (*n=*100), China, Namibia, Thailand and the United States; residing in jurisdictions with HIV criminalization laws was independently associated with reduced ART adherence [131]. Possible mechanisms for this observation include fear of stigma, discrimination, or forced disclosure associated with continued use of ART in the climate of criminalization. When data were limited to North American participants (*n=*1873; 27% women), logistic regression revealed a significant positive association between self-reported ART adherence and residing in jurisdictions where HIV non-disclosure is criminalized, but no significant association between adherence and residing in locations where HIV transmission/exposure is criminalized [132]. Possible pathways to explain these discordant findings are lacking. In particular, the small proportion of women, transgender individuals and minority groups in this study, and an overrepresentation of participants from the United States (*n=*1673), lead the authors to caution against reliably generalizing the findings to these populations and to settings outside the United States [132].

## Limitations of existing literature

We identified only two Canadian studies that explored the effect of HIV criminalization on healthcare engagement specifically among WLWH. Similarly, there is a dearth of literature evaluating the impact of HIV criminalization on the healthcare engagement of individuals who already face the challenge of living in highly criminalized environments (including injection drug users or sex workers), who may face unique barriers to HIV disclosure [55] and engagement with criminal justice and healthcare systems [133]. Most Canadian evidence evaluating the impact of HIV criminalization on healthcare engagement emerges from studies conducted in Ontario, where the majority of Canadian non-disclosure prosecutions have occurred [39]. Although the federal parliament sets criminal law, its application varies in different provincial and territorial jurisdictions. Furthermore, key affected populations and healthcare provision and delivery also vary across Canada [41, 134]. As such, the effect of HIV criminalization on healthcare engagement may vary across health jurisdictions. Finally, the majority of Canadian studies included in this review were conducted before the 2012 SCC ruling, thus the effect of recent judicial rulings on the healthcare engagement of PLWH remains undefined.

## Conclusions

Our comprehensive review of the evidence suggests that the criminalization of HIV non-disclosure may represent a structural barrier to healthcare engagement for some Canadian PLWH, discouraging access to HIV testing and linkage to HIV care services required to achieve viral suppression, which is important to promote both individual and population health benefits. We identified several key mechanisms through which HIV criminalization may compromise healthcare engagement, including provoking fears relating to the exposure of confidential medical information, and increasing clinical surveillance and perceived stigma from healthcare providers and the public. This review also presents evidence to suggest that the criminalization of HIV non-disclosure may influence the clinical care provided by healthcare providers, due to uncertainty around HIV non-disclosure case law and tensions between professional standards of healthcare and legal expectations.

Although the incidence of criminal charges for HIV non-disclosure to sexual partners among Canadian WLWH is low [39], our review suggests that the *threat* of criminal charges combined with a heightened perception of stigma and surveillance may alter the environment within which women navigate engagement with healthcare services. Expansion of routine HIV testing [135] and TasP strategies to meet ambitious UNAIDS treatment targets [100], in addition to the use of evidence from HIV phylogenetic analyses in criminal trials of suspected HIV transmission in Canada and other international settings [136–138], may further reinforce the perception of heightened clinical surveillance reported by PLWH in the current legal climate.

Our review identified only two studies specifically evaluating the impact of criminalization of HIV non-disclosure on the healthcare engagement of Canadian WLWH [91, 125]. This is a concern given the growing number of WLWH in Canada, among whom marginalized women are overrepresented [139]. WLWH may experience gender-specific challenges when navigating healthcare engagement within an environment shaped by the criminalization of HIV non-disclosure, due to antenatal HIV testing protocols [102] and unique sexual and reproductive healthcare needs [140]. The evidence reviewed here suggests that the climate of criminalization may exacerbate gendered barriers to healthcare engagement [65–75], particularly among the most marginalized women who already face significant barriers to healthcare engagement [139].

There is a critical need for further research evaluating the barriers to healthcare engagement among WLWH in an environment shaped by HIV criminalization. Capturing the voices of marginalized women who are disproportionately affected by HIV or underserved by health services is vital to fully appreciate the complex interplay between social factors, medical priorities, sexual and reproductive desires, and legal concerns in the decision to engage with health services. Addressing these critical knowledge gaps will inform future public health initiatives to educate and support Canadian WLWH in the current legal climate, with the ultimate aims of optimizing retention in HIV care and bolstering the case against HIV criminalization.

## Appendix

**Appendix: Table 1:** Studies presenting data on the impact of the criminalization of HIV non-disclosure on healthcare engagement of people living with HIV that were discussed in this literature review

Publications	Study setting	Methodology	Study population and sample size	Data collection	Study outcomes relevant to literature review objectives
**Data collection commenced before R v. Mabior ruling**.					
The problem of “significant risk”: exploring the public health impact of criminalizing HIV non-disclosure (2011) [4]. HIV non-disclosure and the criminal law: establishing policy options for Ontario (2010) *(Analysis among 25 service providers and 28 people living with HIV)* [39].	Ontario, Canada	Qualitative	56 participants: 28 service providers and 28 people living with HIV (*n*=11 women).	Individual semi-structured interviews and focus group discussions from January to September 2010 in Toronto, Ottawa and Hamilton.	Impact of criminal law on: • Linkage and retention in HIV care
How criminalization is affecting people living with HIV in Ontario (2012) [80]. Impacts of criminalization on everyday lives of people living with HIV in Canada (2014) *(qualitative results only)* [81].	Ontario, Canada	Mixed methods	Qualitative component: 122 People living with HIV (*n*=19 women) drawn from the Ontario HIV Treatment Network Cohort Study (OCS). Quantitative component: 925 people living with HIV enrolled in OCS or Positive Spaces, Healthy Places cohort study (PSHP) (*n*=216 women).	In-depth interviews with 122 people living with HIV in Ontario. Participant response to specific questions on HIV and the law in the OCS and PSHP questionnaires between 2009 and 2010.	Impact of criminal law on: • HIV testing • Linkage and retention in HIV care
Impact of prosecution of non-disclosure of HIV status on attitudes and behaviour of HIV negative and HIV positive men who have sex with men (MSM) in Toronto, Ontario (2013) [97].	Ontario, Canada	Quantitative	442 sexually active MSM (292 HIV-positive and 150 HIV-negative).	Detailed questionnaire completed at a Toronto medical clinic between 2010 and 2012.	Impact of criminal law on: • HIV diagnosis and testing
Nondisclosure prosecutions and population health outcomes: examining HIV testing, HIV diagnoses, and the attitudes of men who have sex with men following nondisclosure prosecution media releases in Ottawa, Canada (2013) [95].	Ontario, Canada	Mixed methods	Qualitative: 27 MSM (12 HIV-positive and 15 HIV-negative).	Investigated trends in monthly HIV tests among MSM, conducted in the Ottawa Public Health region from 2008 to 2011. In depth, semi-structured qualitative interviews conducted following a high profile nondisclosure prosecution media release in May 2010.	Impact of criminal law on: • HIV diagnosis and testing • Linkage and retention in HIV care
Sexual practices and STI/HIV testing among gay, bisexual and men who have sex with menin Ottawa, Canada: examining nondisclosure prosecutions and HIV prevention (2013) [98].	Ontario, Canada	Quantitative	Convenience sample of 721 sexually active HIV positive and negativegay, bisexual, and other MSM in Ottawa.	Anonymous surveys self-administered in 14 venues across Ottawa, including bath houses, medical clinics, gay bars and HIV/AIDS organizations.	Impact of criminal law on: • HIV diagnosis and testing • Linkage and retention in HIV care
Nondisclosure prosecutions and HIVprevention: results from an Ottawa-based gay men's sex survey (2013) *(Analysis among 441 participants)* [99].					
Male Call Canada (2013) [89].	National, Canada	Quantitative	Nationally representative sample of 1235 HIV positive and negative MSM.	Cross-sectional national telephone survey of MSM from October 2011 to February 2012.	Impact of criminal law on: • HIV diagnosis and testing
HIV and AIDS in Canada: A National Survey Summary Report (2012) [96].	National, Canada	Quantitative	National sample of 2139 people living in Canada aged ≥16 (52% women).	Cross-sectional national telephone and online survey administered in May 2011.	Impact of criminal law on • HIV diagnosis and testing
HIV Criminalization and Nursing Practice (2012) [119].	Ontario, Canada	Qualitative	47 service providers, working in nursing, medicine, law and social work in Ontario.	8 focus group discussions of 6 individuals facilitated by nursing students, conducted during a meeting on HIV Criminalization & Nursing Practice in 2011.	Impact of criminal law on • Linkage and retention in HIV care
The impact of HIV/AIDS criminalization on awareness, prevention and stigma: a qualitative analysis on stakeholder's perspectives in Ontario, Canada (2012) [94].	Ontario, Canada	Qualitative	Purposive sample of 14 stakeholders from Ontario, including 5 executive directors of HIV organizations, 5 front-line employees of HIV organizations, 4 policy/content experts.	Semi-structured interviews conducted over the telephone.	Impact of criminal law on • HIV diagnosis and testing
The criminalization of HIV non disclosure: what does it mean for policy and practice for a women-specific ASO? (2015) [92].	British Columbia, Canada	Qualitative	60 women living with HIV in Vancouver.	6 focus groups conducted at an AIDS Service Organization in Vancouver (Positive Women's Network), between 2010 and 2014.	Impact of criminal law on • HIV diagnosis and testing in HIV care
The Sero Project: National Criminalization Survey Preliminary Results, (2012) [131].	National, United States	Quantitative	2076 people lving with HIV across the United States (13% women).	Online National HIV Criminalization Survey administered between June and July 2012.	Impact of criminal law on • Access and adherence to ART
Freedom to adhere: the complex relationship between democracy, wealth disparity, social capital and HIV medication adherence in adults living with HIV (2012) [132]. Associations between the legal context of HIV, perceived social capital, and HIV	International	Quantitative	2149 people living with HIV (29% women) from 16 sites across Canada, China, Namibia, Thailand, the United States including Puerto Rico (*n*=100 participants from Canada).	Cross-sectional survey data from the international nursing collaborative study. Drawn from convenience sample of people living with HIV recruited from infectious disease clinics and AIDS Service Organizations	Impact of criminal law on • Access and adherence to ART
antiretroviral adherence in North America (2013) *(Analysis among North American participants)* [133].			Sub-analysis conducted among 1873 people living with HIV (27% women) from Canada, United States including Puerto Rico.	between August 2009 and January 2012. Data on HIV criminal law were drawn from the published literature.	
**Data collection commenced after R v. Mabior ruling**.					
The impact of criminalization of non-disclosure of HIV positive status on racialized communities (2013) [118].	Ontario, Canada	Qualitative	62 participants, including: African/Black men and women living with HIV, mental health service providers, individuals working in community agencies, academics, lawyers, government officers.	Semi-structured interviews and Arts-based research methods conducted in the Greater Toronto Area.	Impact of criminal law on: • Linkage and retention in HIV care
“Using a stick to beat people down”: perceptions of criminalization of HIV non-disclosure and testing practices among men in Nova Scotia (2014) [93].	Newfoundland, Canada	Qualitative	Six health professionals who work with men living with HIV in Nova Scotia.	Two focus groups held in Nova Scotia.	Impact of criminal law on: • HIV diagnosis and testing
Sexuality, prevention work & the criminalization of non-disclosure of HIV (2014) [120].	Ontario, Canada	Qualitative	40 people living with HIV and 15 prevention workers.	Qualitative interviews conducted in Toronto.	Impact of criminal law on: • Linkage and retention in HIV care
The effect of R v. Mabior on HIV/AIDS service provision (2014) [121].	Ontario, Canada	Qualitative	15 HIV service providers, working in HIV prevention and supportive services.	Semi-structured interviews in Toronto.	Impact of criminal law on: • Linkage and retention in HIV care
Judging mothers: criminalization's creep into the health and social care of HIV-positive mothers (2014) [126].	Ontario, Canada	Qualitative	77 pregnant women living with HIV from Ontario (participants of the HIV Mothering Study).	Interviews conducted in Ontario with women in their 3rd trimester, and at 3 and 12 months postpartum.	Impact of criminal law on: • Linkage and retention in HIV care
Discussing the Limits of Confidentiality: The Impact of Criminalizing HIV Nondisclosure on Public Health Nurses’ Counselling Practices (2014) [122]. Examining public health nurses’ documentary practices: the impact of criminalizing HIV non- disclosure on inscription styles (2015) [123].	Ontario, Canada	Qualitative	Purposive sample of 30 nurses with experience working as HIV case managers from four public health departments.	One-on-one semi-structured interviews in Ontario.	Impact of criminal law on: • Linkage and retention in HIV care
